# Ocular adverse events associated with anti-VEGF therapy: A pharmacovigilance study of the FDA adverse event reporting system (FAERS)

**DOI:** 10.3389/fphar.2022.1017889

**Published:** 2022-11-18

**Authors:** Pan Ma, Xinmei Pan, Ruixiang Liu, Ya Qu, Linli Xie, Jiangchuan Xie, Liya Cao, Yongchuan Chen

**Affiliations:** ^1^ Department of Pharmacy, The First Affiliated Hospital of Army Medical University, Chongqing, China; ^2^ Southwest Hospital/Southwest Eye Hospital, Third Military Medical University (Army Medical University), Chongqing, China

**Keywords:** adverse events, pharmacovigilance, ranibizumab, aflibercept, brolucizumab, safety signals

## Abstract

**Background:** The purpose of this study is to identify and characterize ocular adverse events (AEs) that are significantly associated with anti-VEGF drugs for treatment of neovascular age-related macular degeneration and compare the differences between each drug, and provide clinical reference.

**Methods:** Ocular AEs submitted to the US Food and Drug Administration were analyzed to map the safety profile of anti-VEGF drugs. The Pharmacovigilance tools used for the quantitative detection of signals were reporting odds ratio and bayesian confidence propagation neural network.

**Results:** A total of 10,608,503 AE reports were retrieved from FAERS, with 20,836 for ranibizumab, 19,107 for aflibercept, and 2,442 for brolucizumab between the reporting period of Q1, 2004 and Q3, 2021. We found and analyzed the different AEs with the strongest signal in each drug—ranibizumab-macular ischaemia (ROR = 205.27, IC-2SD = 3.70), retinal pigment epithelial tear (ROR = 836.54, IC-2SD = 7.19); aflibercept-intraocular pressure increased (ROR = 31.09, IC-2SD = 4.61), endophthalmitis (ROR = 178.27, IC-2SD = 6.70); brolucizumab-retinal vasculitis (ROR = 2930.41, IC-2SD = 7.47) and/or retinal artery occlusion (ROR = 391.11, IC-2SD = 6.10), dry eye (ROR = 12.48, IC-2SD = 2.88).

**Conclusion:** The presence of AEs should bring clinical attention. The use of anti-VEGF drugs should be based on the patient’s underlying or present medical condition to reduce any adverse event associated with the treatment.

## Introduction

Age-related macular degeneration (AMD) is an acquired disease of the macula, a progressive visual impairment caused by late-onset neurodegeneration of the photoreceptor-retinal pigment epithelial complex (Waseem and Sanaa, 2017). AMD is the leading cause of severe and irreversible vision loss for people aged 55 years and over in developed countries (Congdon et al., 2004), and it becomes more serious with the aging of population, with an anticipated rise to 288 million cases worldwide by year 2040 (Wong et al., 2014). AMD can be classified into dry and neovascular (wet) according to the absence or presence of new choroidal blood vessels that invade the retina, respectively (Ambati and Fowler, 2012). Anti-VEGF drugs have set the benchmark in the treatment of neovascular AMD (Velez-Montoya et al., 2013), due to its ability to suppress choroidal neovascularization (CNV), reduce retinal fluid leakage and improve visual impairment (Campochiaro et al., 2016).

Currently, intravitreal injection of anti-VEGF drugs includes ranibizumab, aflibercept, off-label bevacizumab, and brolucizumab (Arepalli and Kaiser, 2021). Ranibizumab is a recombinant humanized IgG1monoclonal Fab fragment, which binds to and inhibits the biologic activity of human vascular endothelial growth factor A (VEGF-A). It can improve average visual acuity, and ameliorate classic CNV remarkably (Brown et al., 2009). Aflibercept is a recombinant fusion protein with the Fc portion, has high affinity to all VEGF-A and VEGF-B isoforms and placental growth factors. It was approved by the FDA in 2011 to treat neovascular AMD (Heier et al., 2012). Bevacizumab originally developed as a chemotherapeutic drug, mainly for the treatment of colorectal cancer, non-small cell lung cancer and other forms of cancer. Its off-label use for the treatment of neovascular AMD, due to the lack of specificity to conditions associated with inhibition of VEGF, has been linked to the incidence of serious AEs and thus, has not been approved by the FDA (Grzybowski et al., 2018).

The new anti-VEGF drug brolucizumab is composed of a single-chain antibody fragment structure, which is the smallest anti-VEGF antibody tested in humans and can inhibit all isoforms of VEGF-A (Holz et al., 2016). The HAWK and HARRIER clinical trials reached the primary end point of noninferiority in best corrected visual acuity after the comparison of brolucizumab and aflibercept and thus, approved by the FDA and European Medical Agency in 2019 and 2020 respectively. Phase III clinical trials are well underway in China (Dugel et al., 2020).

Although anti-VEGF drugs are currently recognized as the first-line treatment for neovascular AMD, repeated injections of anti-VEGF drugs can still cause some ocular complications, such as eye pain (Biagi et al., 2014), conjunctival hemorrhage etc. (Dugel et al., 2020). Due to the small difference in the efficacy of the three drugs (Heier et al., 2012; Dugel et al., 2020), clinicians and patients may pay more attention to safety issues. The overall safety of these drugs is satisfactory, but literature review found that there are differences in AEs reported by different drugs. Although they are available, the absence of systematic reports including comparisons of adverse reactions of these drugs in the literature give no conclusive summary of AEs.

Adverse events spontaneous reporting system is currently one of the most important methods in monitoring the safety of medicinal products. FDA Adverse Event Reporting System (FAERS) is a public database designed to support the FDA’s post-marketing safety surveillance program for drug and therapeutic biologic products through a system of spontaneous reports by consumers, health professionals, drug manufacturers, and other non-healthcare workers. Based on the needs of clinical, rational and precise drug use and protection of patients’ rights and interests, we evaluated and compared the AE reports of anti-VEGF drugs using FAERS database. Findings of this study create real-world evidence for risk signal detection and guide future comparative effectiveness and post-marketing surveillance research for anti-VEGF drugs.

## Methods

### Data source

The pharmacovigilance tools used in this study to extract data is OpenVigil, an experimental research application, which availed researchers of directly extracting structured AE report information from the FAERS database through the docking application program interface (API). With the additional drug mapping and duplicate detection functionality, OpenVigil is used in many pharmacovigilance studies. We performed a retrospective pharmacovigilance study based on data from Q1 of 2004 to Q3 of 2021 in the FAERS database. AEs in the FAERS are coded by the preferred-terms level of the Medical Dictionary for Regulatory Activities (MedDRA) classification. Due to a large number of preferred terms and their lack of specificity, Standardized MedDRA Queries (SMQs) were developed. SMQs are standard sets of MedDRA terms that are related to the same medical condition, thereby facilitating data retrieval and signal detection.

### Ethics approval

De-identified public data was used in this study, not requiring any form of ethics approval.

### Adverse events and drug identification

Reports involving three kinds of anti-VEGF drugs for neovascular AMD treatment (including ranibizumab, aflibercept and brolucizumab) were identified using text string searches for each drug by brand and generic names through the FDA public database during the data mining process. Then, we extracted AEs marking “ranibizumab”, “aflibercept”, “brolucizumab” and brand name “Lucentis”, “Byooviz”, “Susvimo”, “Zaltrap”, “Eylea”, “Beovu” as the primary suspected object. AEs can be specified at different levels of the MedDRA terminology.

**FIGURE 1:**
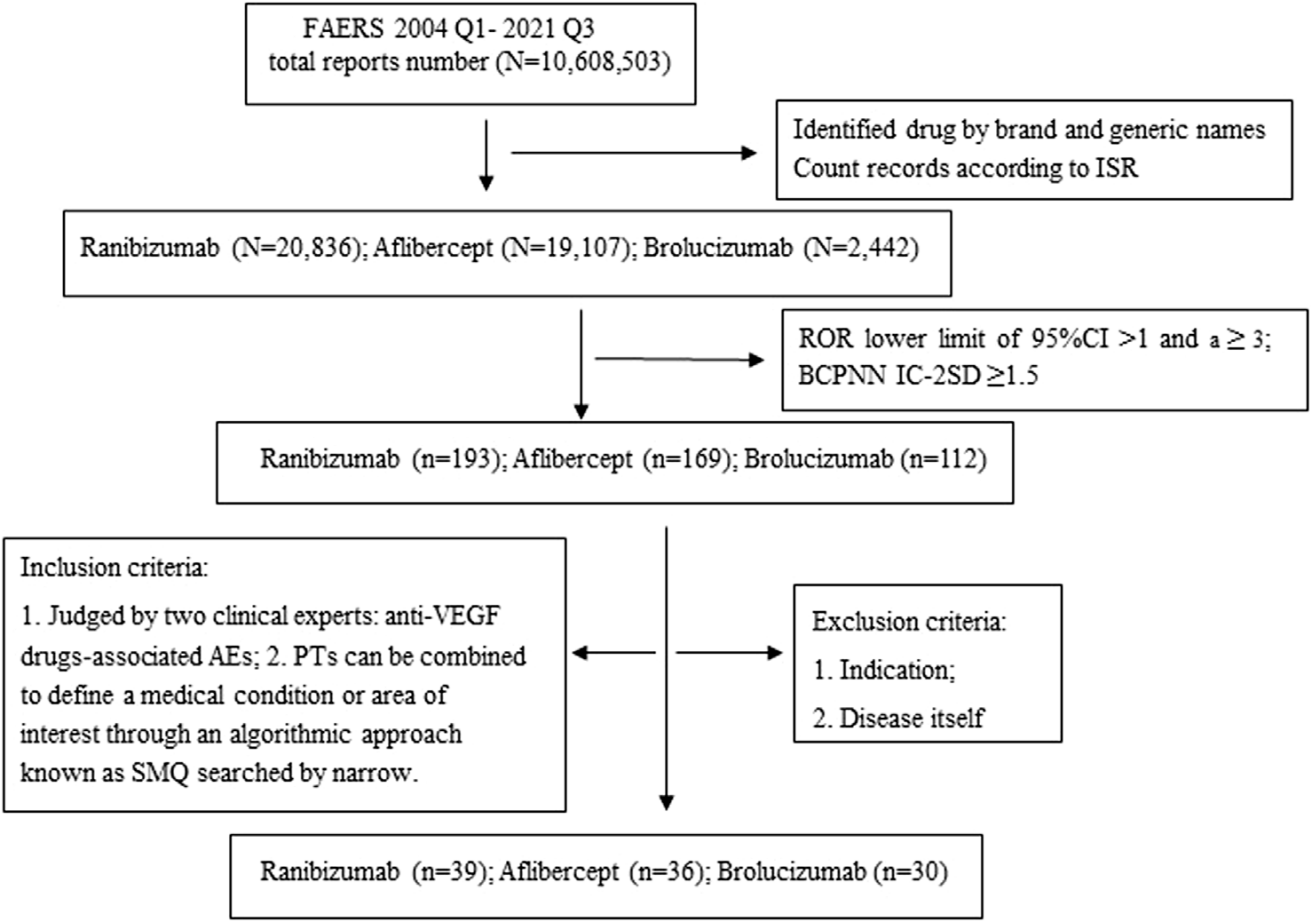
The workflow of data mining. Abbreviations: N, Total number of adverse drug events; n, Safety signals; ISR, Number that uniquely identifies an AERS report; PTs, Preferred Terms; SMQ, Standardized MedDRA Queries.

We searched with preferred term (PT) as primary term and counting records according to Individual Safety Reports (ISR). As a result, the safety profile of each of the anti-VEGF drugs was examined through SMQ analysis. Two researchers, including a chief pharmacist and a professor of Ophthalmology classified the AE reports in terms of SMQs and collected clinical characteristics of the patient, including gender, age, and AE outcomes, respectively. Unexpected adverse drug reaction was defined as any significant AE that was uncovered and was not listed in the FDA drug labelling. To minimize the existence of an “indication bias” (i.e., the indication for which the prescribed drug is reported as an AE), PTs and SMQs associated with AMD-related signs and complications were removed for analysis. The workflow of the study as shown in Figure 1.

### Data mining

One of the most frequently used methods of safety signal detection is disproportionality analysis, which consisted of two categories: Frequentist Statistics and Bayesian Statistics. Frequentist Statistics included reporting odds ratio (ROR), and proportional reporting ratio (PRR). Bayesian Statistics on the other hand, included bayesian confidence propagation neural network (BCPNN) and multi-item gamma poisson shrinker (MGPS). The frequentist method had its characteristics: the sensitivity of frequency method was high, but it was easy to produce false positive signals when the number of reports was small. The specificity of Bayesian method was good; however, the signal detection time was relatively delayed. In order to minimize the result bias caused by using a certain algorithm alone, two methods, ROR and BCPNN, were used for signal detection in this study. When both algorithms were positive, they were judged as suspicious signals. The ratio imbalance measurement algorithm was shown in Table 1. The principle of disproportionate measure and standard of signal detection were shown in Table 2.

**TABLE 1:** Ratio imbalance measurement algorithm.

Item	Reports with the target AEs	All other AEs	Total
Reports with the target drug	a	b	a+b
All other drugs	c	d	c + d
Total	a+c	b + d	a+b + c + d

**TABLE 2:** Principle of dis-proportionality measure and standard of signal detection.

Algorithms	Calculation formula	Criteria
ROR	$ROR=\frac{a/c}{b/d}=\frac{ad}{bc}$	(1) a ≥3;
	$95\%CI=e^{\ln\left(ROR\right)\pm 1.96\sqrt{\frac{1}{a}+\frac{1}{b}+\frac{1}{c}+\frac{1}{d}}}$	(2) 95%CI > 1
BCPNN	$E\left(IC\right)=\log_{2}\frac{\left(C_{xy}+\gamma_{11}\right)\left(C+\alpha \right)\left(C+\beta \right)}{\left(C+\gamma \right)\left(C_{x}+\alpha_{1}\right)\left(C_{y}+\beta_{1}\right)}$	(1) a ≥3;
		(2) IC-2SD > 0;
		(3) IC-2SD ≥ 1.5 (medium and strong signals)
	$V\left(IC\right)=1/{\left(In2\right)}^{2}\left\{\left(\frac{C-C_{xy}+\gamma -\gamma_{11}}{\left(C_{xy}+\gamma_{11}\right)\left(1+C+\gamma \right)}\right)+\left(\frac{C-C_{x}+\alpha -\alpha_{1}}{\left(C_{x}+\alpha_{1}\right)\left(1+C+\alpha \right)}\right)+\left(\frac{C-C_{y}+\beta -\beta_{1}}{\left(C_{y}+\beta_{1}\right)\left(1+C+\beta \right)}\right)\right\}$	
	$\gamma =\gamma_{11}\frac{\left(C+\alpha \right)\left(C+\beta \right)}{\left(C_{x}+\alpha_{1}\right)\left(C_{y}+\beta_{1}\right)}$	
	$IC-2SD=E\left(IC\right)-2\sqrt{V\left(IC\right)}$	
	$\alpha_{1}=\beta_{1}=1;\alpha =\beta =2;\gamma_{11}=1;$	
	$C=a+b+c+d;C_{x}=a+b;C_{y}=a+c;C_{xy}=a$	

Abbreviations: ROR, Reporting odds ratio; BCPNN, Bayesian confidence propagation neural network; CI, Confidence Interval; IC, Information Component.

### Statistical analysis

Using the ROR and BCPNN, when the lower limit of the 95% confidence interval (CI) of ROR exceeds 1.0 and the information component value minus two standard deviations (IC-2SD) of BCPNN is greater than zero, with at least three records, it is an indication of a safety signal. In addition, the time scan map of safety signal was shown reflecting the trend of a drug paired with AE in FAERS based on the IC 95% CI. When the time scan map is in a steady upward trend and the 95% CI is narrowed, the signal is stable and the association between the drug and the AE is strong. According to BCPNN signal strength standard, medium and strong signals with signal value IC-2SD ≥ 1.5 were selected for analysis and discussion (Sharwan and Bhaswat, 2015). All analyses were performed using Microsoft EXCEL 2019. Figures were illustrated using GraphPad Prism (v8.2) or R (v4.1.2).

## Results

In this study, data mining was performed to obtain the safety signals of anti-VEGF drugs from Q1 of 2004 to Q3 of 2021. A total of 10,608,503 AE reports were retrieved from FAERS, with 20,836 for ranibizumab, 19,107 for aflibercept, and 2,442 for brolucizumab. Based on the geographical perspective, majority of the reports were from America. In gender, reports for females were approximately 10%–20% more than males for both ranibizumab and brolucizumab. For aflibercept, the highest tallied reports fell under unknown gender. For age composition, bulk of the reports were from people aged 50–79 across all three drugs, followed closely by respondents aged 80 and above. The serious outcomes related to aflibercept accounted for a relatively high proportion (11,356 cases, 59.4%). On the other hand, hospitalization, disability and other life-threatening events were unlikely as the numbers were relatively low. The demographic characteristics of AE reports associated with Anti-VEGF drugs are shown in Table 3.

**TABLE 3:** Characteristics of reports associated with Anti-VEGF from Q1 of 2004 to Q3 of 2021.

	Ranibizumab (%)	Aflibercept (%)	Brolucizumab (%)
Number of events	20836	19107	2442
Gender			
Female	9855 (47.3)	1463 (7.7)	1298 (53.2)
Male	7677 (36.8)	1529 (8.0)	808 (33.1)
Unknown	3304 (15.9)	16115 (84.3)	336 (13.8)
Age			
<18	52 (0.2)	3 (0)	1 (0)
18–49	316 (1.5)	165 (0.9)	4 (0.2)
50–79	4702 (22.6)	1644 (8.6)	674 (27.6)
≥80	4056 (19.5)	712 (3.7)	577 (23.6)
Unknown	11710 (56.2)	16583 (86.8)	1186 (48.6)
Serious outcomes			
Death	4958 (23.8)	7947 (41.6)	150 (6.1)
Disability	596 (2.9)	1269 (6.6)	107 (4.4)
Life-threatening	309 (1.5)	143 (0.7)	5 (0.2)
Hospitalization	3572 (17.1)	1997 (10.5)	106 (4.3)
Total	9435 (45.3)	11356 (59.4)	368 (15.1)
Reporter country			
USA	7497 (36.0)	12731 (66.6)	1143 (51.0)
Japan	1296 (6.2)	871 (4.6)	232 (10.3)
Germany	713 (3.4)	438 (2.3)	92 (4.1)
Other countries	11330 (54.4)	5067 (26.5)	775 (34.6)

A total of 43 moderate to strong signals with an IC-2SD ≥ 1.5 were identified under 3 kinds of anti-VEGF drugs in Table 4. Some were presented in the instructions while marked signals in the table were found from FAERS database. For instance, macular ischaemia was not indicated in ranibizumab’s drug label and yet, was found to have a strong signal. The following is classified as the top AEs in each drug: ranibizumab-macular ischaemia, retinal pigment epithelial tear (RPE tear); aflibercept-intraocular pressure increase, endophthalmitis; brolucizumab-retinal vasculitis and/or retinal vascular occlusion, dry eye. We listed the moderate to strong signals in Table 4, and selected three PTs with the strongest safety signals of each drug that are more clinically concerned to draw IC time scan picture.

**TABLE 4:** Moderate and strong signals of anti-VEGF drugs in ocular adverse events.

SMQs/PTs	Ranibizumab			Aflibercept			Brolucizumab		
	N	ROR (95%CI)	IC (IC-2SD)	N	ROR (95%CI)	IC (IC-2SD)	N	ROR (95%CI)	IC (IC-2SD)
Retinal disorders									
Retinal pigment epithelial tear	356	836.54 (706.72, 990.22)	7.39 (7.19)	55	58.67 (44.42, 77.50)	4.78 (4.37)	10	77.05 (41.18, 144.16)	3.28 (2.40)
Detachment of retinal pigment epithelium	292*	440.01 (376.16, 514.69)	7.03 (6.82)	63	61.35 (47.27, 79.61)	4.90 (4.52)	18	127.85 (79.88, 204.63)	4.05 (3.38)
Vitreous haemorrhage	333*	133.70 (118.43, 150.94)	6.32 (6.14)	157*	60.00 (50.86, 70.78)	5.33 (5.09)	14*	38.10 (22.48, 64.57)	3.45 (2.70)
Retinal haemorrhage	660*	97.72 (89.83, 106.31)	6.16 (6.04)	307*	44.37 (39.47, 49.88)	5.17 (5.00)	72	77.97 (61.55, 98.76)	5.21 (4.87)
Retinal scar	122*	377.92 (298.95, 477.77)	6.30 (5.98)	25*	52.95 (35.12, 79.83)	4.10 (3.51)	/	/	/
Macular hole	117*	95.96 (78.73, 116.97)	5.59 (5.30)	101*	88.06 (71.35, 108.68)	5.45 (5.14)	/	/	/
Vitreous floaters	401	50.89 (45.89, 56.44)	5.36 (5.21)	386	53.28 (47.96, 59.20)	5.41 (5.26)	453	599.32 (538.71, 666.74)	7.80 (7.65)
Subretinal fibrosis	59*	375.82 (268.40, 526.22)	5.56 (5.11)	/	/	/	6*	196.41 (86.59, 445.50)	2.76 (1.64)
Retinal tear	132*	62.55 (52.18, 74.98)	5.30 (5.03)	21	9.79 (6.36, 15.08)	2.79 (2.17)	9	32.64 (16.92, 62.95)	2.97 (2.05)
Retinal ischaemia	56*	89.16 (67.10, 118.47)	5.03 (4.62)	18*	27.89 (17.37, 44.79)	3.50 (2.82)	23*	285.66 (187.01, 436.37)	4.46 (3.85)
Retinal detachment	261	30.46 (26.86, 34.53)	4.69 (4.50)	119	14.58 (12.15, 17.50)	3.67 (3.40)	8	122.31 (60.51, 247.23)	3.08 (2.10)
Vitreous detachment	72	37.96 (29.87, 48.25)	4.58 (4.23)	31	17.07 (11.94, 24.41)	3.48 (2.96)	9	38.09 (19.74, 73.50)	3.01 (2.09)
Retinal artery occlusion	76*	29.52 (23.41, 37.21)	4.37 (4.03)	74*	31.31 (24.76, 39.58)	4.42 (4.08)	110	391.11 (320.55, 477.19)	6.39 (6.10)
Retinal depigmentation	27*	112.60 (74.20, 170.88)	4.43 (3.84)	9*	35.64 (18.16, 69.95)	2.98 (2.03)	/	/	/
Macular ischaemia	23*	205.27 (126.46, 333.18)	4.37 (3.70)	/	/	/	/	/	/
Retinal vascular thrombosis	24*	36.12 (23.87, 54.66)	3.87 (3.27)	/	/	/	/	/	/
Vitreous haze	17*	154.38 (89.70, 265.70)	3.97 (3.22)	29*	365.83 (228.87, 584.76)	4.73 (4.10)	25	2285.46 (1407.06, 3712.23)	4.68 (4.02)
Retinal vasculitis	28*	23.82 (16.30, 34.81)	3.70 (3.15)	51*	49.29 (37.00, 65.65)	4.61 (4.19)	237	2930.41 (2480.17, 3462.39)	7.70 (7.47)
Photopsia	68*	10.81 (8.50, 13.75)	3.21 (2.86)	28*	4.79 (3.30, 6.94)	2.07 (1.53)	35*	47.60 (34.03, 66.57)	4.36 (3.87)
Vitreal cells	/	/	/	28*	361.41 (224.49, 581.84)	4.68 (4.05)	37*	4799.11 (3007.42, 7658.20)	5.22 (4.64)
Ocular infections									
Endophthalmitis	590	109.11 (99.74, 119.36)	6.26 (6.13)	805	178.27 (164.51, 193.19)	6.82 (6.70)	38	49.60 (35.94, 68.46)	4.45 (3.98)
Vitritis	97	67.92 (54.93, 83.99)	5.22 (4.91)	237*	225.79 (194.06, 262.71)	6.58 (6.37)	196	1469.04 (1244.90, 1733.54)	7.37 (7.13)
Hypopyon	41*	44.51 (32.34, 61.26)	4.39 (3.93)	66*	82.67 (63.81, 107.10)	5.12 (4.75)	18*	160.07 (99.84, 256.63)	4.09 (3.41)
Eye infection	160*	19.64 (16.76, 23.01)	4.08 (3.85)	127*	16.85 (14.12, 20.12)	3.86 (3.60)	18*	18.26 (11.47, 29.06)	3.25 (2.58)
Blepharitis	22	7.77 (5.10, 11.84)	2.57 (1.96)	17*	6.53 (4.04, 10.53)	2.31 (1.62)	/	/	/
Glaucoma									
Ocular hypertension	102*	68.17 (55.42, 83.85)	5.25 (4.95)	62*	42.87 (33.10, 55.53)	4.62 (4.24)	7*	35.49 (16.85, 74.75)	2.74 (1.71)
Intraocular pressure increased	310	21.58 (19.24, 24.19)	4.27 (4.10)	402	31.09 (28.10, 34.41)	4.76 (4.61)	80	47.01 (37.58, 58.81)	4.87 (4.54)
Glaucoma	182*	9.85 (8.50, 11.42)	3.20 (2.98)	100*	5.83 (4.79, 7.11)	2.46 (2.17)	27*	12.32 (8.43, 18.01)	3.12 (2.57)
Lens disorders									
Cataract	430	9.41 (8.54, 10.36)	3.16 (3.01)	261	6.14 (5.43, 6.94)	2.56 (2.38)	62	11.47 (8.91, 14.76)	3.27 (2.90)
Posterior capsule opacification	19	90.31 (55.43, 147.14)	4.00 (3.31)	7*	32.61 (15.21, 69.90)	2.70 (1.65)	6*	217.69 (95.79, 494.71)	2.77 (1.64)
Posterior capsule rupture	8*	63.54 (30.46, 132.53)	2.98 (1.96)	/	/	/	/	/	/
Lenticular opacities	9*	23.34 (11.96, 45.54)	2.83 (1.90)	/	/	/	/	/	/
Toxic anterior segment syndrome	/	/	/	20*	24.17 (15.45, 37.83)	3.49 (2.85)	/	/	/
Corneal disorders									
Corneal abrasion	41*	33.83 (24.65, 46.42)	4.19 (3.73)	28*	24.67 (16.89, 36.03)	3.73 (3.18)	/	/	/
Corneal erosion	22*	37.93 (24.59, 58.51)	3.82 (3.20)	10*	18.06 (9.62, 33.91)	2.81 (1.92)	/	/	/
Corneal oedema	35*	14.46 (10.33, 20.24)	3.37 (2.88)	53	24.26 (18.42, 31.95)	4.04 (3.64)	9*	31.18 (16.17, 60.14)	2.95 (2.03)
Keratic precipitates	14*	44.49 (25.76, 76.84)	3.48 (2.70)	36*	144.85 (100.34, 209.09)	4.81 (4.29)	121*	10432.45 (7534.69, 14444.65)	6.87 (6.53)
Corneal opacity	/	/	/	14*	12.58 (7.41, 21.38)	2.81 (2.06)	9*	63.07 (32.62, 121.94)	3.13 (2.20)
Conjunctival disorders									
Conjunctival haemorrhage	70	16.73 (13.18, 21.23)	3.74 (3.39)	53	13.70 (10.43, 17.99)	3.44 (3.04)	/	/	/
Conjunctival hyperaemia	22	6.64 (4.36, 10.11)	2.40 (1.79)	19*	6.24 (3.97, 9.81)	2.29 (1.64)	21	54.52 (35.39, 84.00)	3.98 (3.36)
Conjunctivitis	63*	4.38 (3.42, 5.62)	2.05 (1.68)	/	/	/	/	/	/
Lacrimal disorders									
Lacrimation increased	183	8.04 (6.95, 9.31)	2.92 (2.71)	136	6.48 (5.47, 7.68)	2.62 (2.37)	70	26.57 (20.94, 33.73)	4.25 (3.90)
Dry eye	/	/	/	/	/	/	46*	12.48 (9.32, 16.71)	3.30 (2.88)

Abbreviations: PTs, Preferred Terms; SMQs, Standardised MedDRA Queries; N, Number of target adverse events of target drug.

/indicates that IC-2SD value of the adverse event is less than 1.5.

*indicates that this adverse reaction is not in the instructions.

**FIGURE 2:**
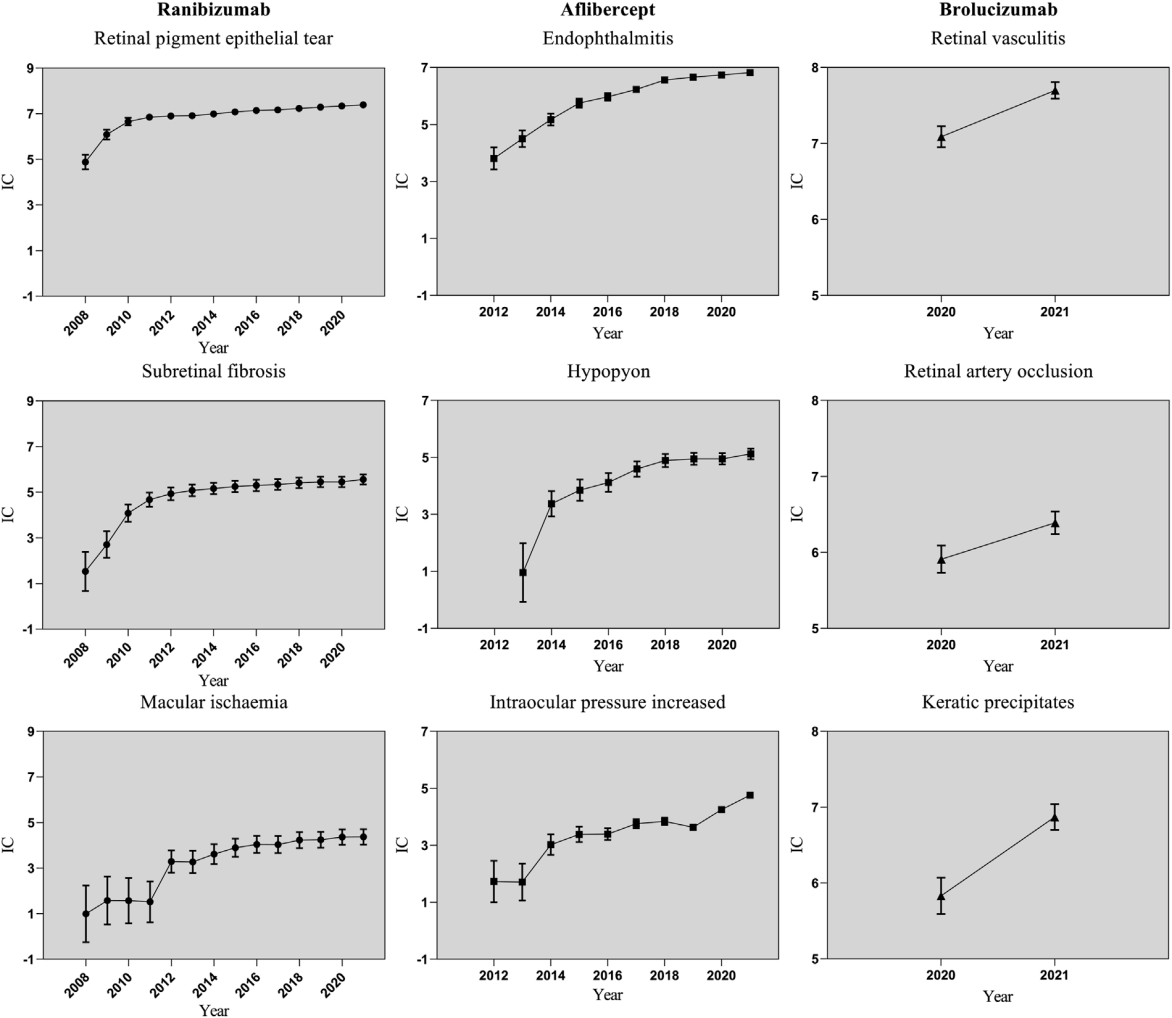
Information component and its 95% credibility interval over time for different types of anti-VEGF-associated ocular adverse events. Abbreviations: ●, Ranibizumab; ■, Aflibercept; ▲, Brolucizumab; IC, information component; CI, credibility interval. The error bars show the 95% credibility interval (CI) of the information component (IC), when the IC curve is steady upward trend and the 95% CI narrowed, the signal is stable and strong association.

In order to investigate the changes of each signal over time, this study drew time scans of safety signals of RPE tear, subretinal fibrosis and macular ischaemia for ranibizumab; endophthalmitis, hypopyon and intraocular pressure increase for aflibercept; and retinal vasculitis, retinal artery occlusion and keratic precipitates for brolucizumab. Each graph shows a steady or upward trend and the confidence interval gradually narrows (as shown in Figure 2), which indicates that the signal is stable and strongly correlated with the use of the anti-VEGF drug. The abscissa was the year of the report, and the ordinate was the Information Component (IC) value. IC values of anti-VEGF drugs induced various AEs from 2008 to 2021. As the years went by, the number of reports increased. Moreover, IC values accumulates gradually and the range of confidence interval continues to narrow across all three anti-VEGF drugs.

**FIGURE 3:**
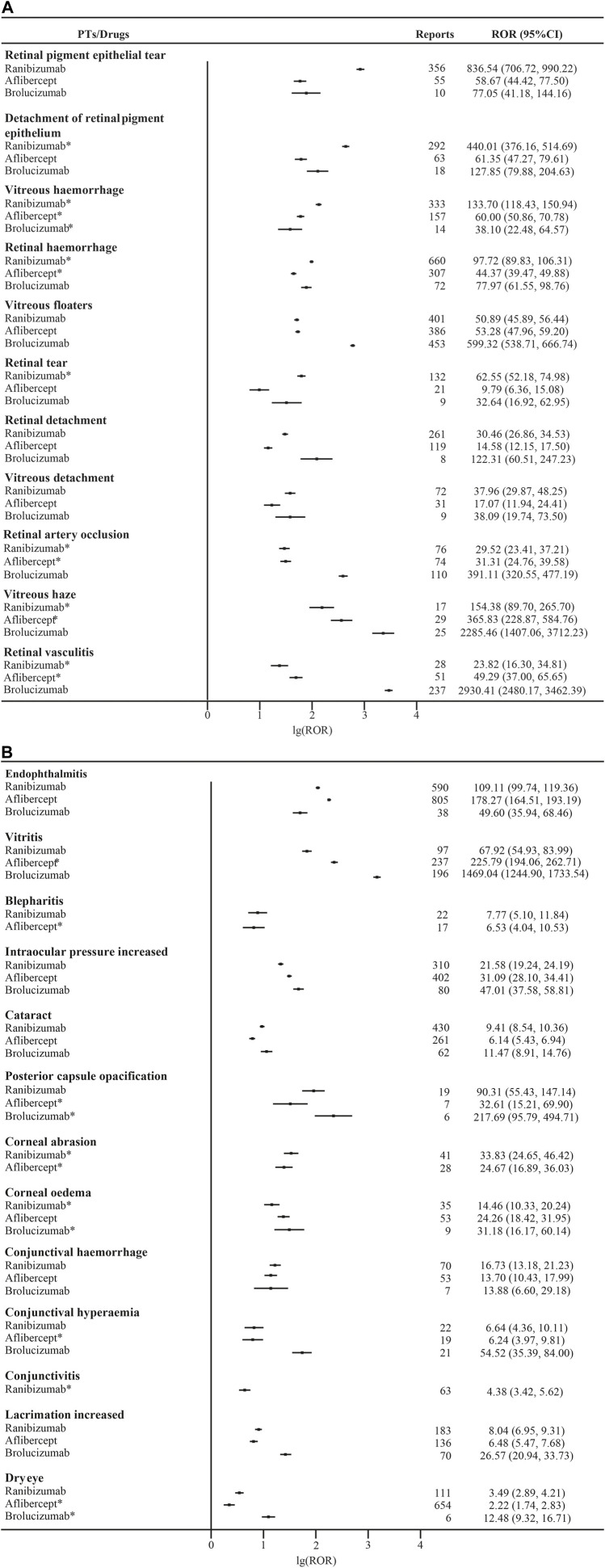
(Continued) Reporting Odds Ratios (RORs) for ocular adverse events associated with anti-VEGF. Abbreviations: **(A)**: Retina-related adverse events; **(B)**: Adverse events unrelated to retinas; 95% CI, 95% confidence interval. * indicates that this adverse reaction is not in the instructions.

All moderate and strong signals associated with anti-VEGF drugs are shown in the Supplementary Data, Supplementary Tables S1–S3. We compared the AE signals mentioned in the instructions and found that the signals of different drugs had their individual characteristics as shown in Figure 3. In general, the manifestation is that AE signals related to retina tallied higher ROR figures than those of retina-unrelated AEs except for vitritis, which totaled 1469.04 RORs for brolucizumab, the highest in its class. In the same category, endophthalmitis had 178.27 RORs for aflibercept. For retina related AEs, brolucizumab collected 391.11 and 2930.41 RORs for retinal artery occlusion and retinal vasculitis respectively while aflibercept had the two lowest RORs for retinal tear at 9.79 and retinal detachment at 14.58. RPE tear ranked the highest ROR of ranibizumab in both groups at 836.54. We found that all three drugs have their individual AEs.

## Discussion

To the best of our knowledge, this study is the first to identify and characterize ocular AEs that are significantly associated with anti-VEGF drugs. Based on the database, we carried out 7 SMQs of ocular related AEs, and put emphasis on those safety signals that were classified as strong signals in the AE reports of anti-VEGF drugs in FAERS. After consulting with ophthalmologists and combining medical knowledge, we analyzed the unexpected adverse drug reactions that may or may not be listed in the instructions but were of clinical concern, and compared the characteristics of different drugs. We found statistically-significant signals for anti-VEGF drugs in the visual system for ranibizumab (macular ischaemia, RPE tear), aflibercept (intraocular pressure increased, endophthalmitis), and brolucizumab (retinal vasculitis and/or retinal vascular occlusion, dry eye). We analyzed the different adverse reactions and they may be due to molecular weight, structure, mechanism of action and pharmacokinetics of the drugs (Avery et al., 2014; Ferro Desideri et al., 2021).

Macular ischaemia has always been a great concern in medical practice. However, ranibizumab drug instructions do not include such, which possess an even greater risk. This study shows a disproportionate association with macular ischemia of ranibizumab, from 2008 to 2021, and the gradual increase was shown in the IC time scan. VEGF has been known to carry the capacity to promote formation of collateral vessels, which is essential for recovery after ischaemic events (Clayton et al., 2008). In addition, the upregulation of VEGF expression in ischaemic retinal conditions can reduce neuroretinal cell apoptosis that may enhance neuroprotection (Kazuaki Nishijima, 2007). As these agents may downregulate normal physiological functions of VEGF, VEGF blockade-induced vasoconstriction in an already compromised macular capillary bed could further increase hypoxic damage with a potentially devastating effect on macular function and visual outcome. Ranibizumab, compared with aflibercept and brolucizumab, blocks all isoforms of VEGF, and has a Fab fragment that penetrates better through all the retinal layers, thus making the effects stronger (Ferrara et al., 2006). Clinicians have noted and closely monitored macular ischemia after initial and subsequent intravitreal ranibizumab treatment and have recommended the addition of dexamethasone therapy if the condition worsens (Verma and Khetan, 2018). If a patient has symptoms related to macular ischemia at baseline, treatment with ranibizumab should be selected with caution.

Post-injection endophthalmitis is a rare but devastating complication after intravitreal injection of anti-VEGF drugs, and can cause significant vision loss (Mccannel, 2011; Fileta et al., 2014). The most common presenting symptom of endophthalmitis is reduced visual acuity, followed by pain/photophobia, redness, floaters, lid swelling and discharge, and the most common signs are vitritis, hypopyon, hyperemia, corneal edema and increase in intraocular pressure (Lyall et al., 2012; Haddock et al., 2014). The main factors, which play a role in intraocular endophthalmitis after anti-VEGF injection are patient-specific, medication-specific and delivery-specific (Anderson et al., 2021). It has been presented that some patients have anti-idiotype antibodies against anti-VEGF antibody (Sanjeewa et al., 2008). This anti-drug antibody (ADA) titers are associated with inflammation, which may cause endophthalmitis (Baumal et al., 2020). Noninfectious contamination (e.g., endotoxins) and administration formulation during drug manufacturing can also lead to endophthalmitis (Heier et al., 2006; Gasparin et al., 2012; Goldberg et al., 2013; Anderson et al., 2021). The anti-VEGF antibody itself may have immunogenic properties, such as the Fc portion interacting with intraretinal Fc receptors, triggering an inflammatory reaction that may cause endophthalmitis (Murinello et al., 2014; Anderson et al., 2021). In addition, protein aggregation or change in conformation may also cause endophthalmitis (Melo et al., 2019; Anderson et al., 2021; Melo et al., 2021), due to the delivery-specific constraints.

A large retrospective research report shows that the incidence of endophthalmitis after aflibercept injection is higher than that of ranibizumab (Souied et al., 2016). We identified significant disproportionality of endophthalmitis and its related signs, such as vitritis, anterior chamber empyema, corneal edema, congestion and floaters in three anti-VEGF drugs, which is consistent with literature reports (Haddock et al., 2014). Physicians must be familiar with the clinical manifestations of endophthalmitis after administration in order to make a prompt diagnosis. It is worth noting that this study has unearthed the safety signal of toxic anterior segment syndrome (TASS) of aflibercept as well. The clinical features of TASS are similar to those of endophthalmitis, except that the time and severity of occurrence are inconsistent. The anterior segment inflammation is severe and usually resulting in hypopyon formation (Sengillo et al., 2020), which should raise clinical concern.

Intravitreal anti-VEGF therapy may have adverse effects on ocular blood flow. Several cases of retinal vasculitis and/or retinal vascular occlusion were reported following the FDA approval of brolucizumab (Baumal et al., 2020; Haug et al., 2020). In fact, our study identified that all three drugs have the same safety signals yet we affirmed that brolucizumab carries the strongest one, which may be due to its small molecular structure and high affinity (Holz et al., 2016) that induce stronger effect on hypersensitivity, endothelial cells and nitric oxide production. Further knowledge on the retinal vasculitis and/or retinal vascular occlusion associated with brolucizumab may help guide clinicians in their clinical decision making moving forward.

Several recent publications have reported RPE tear associated with the use of intravitreal VEGF antagonists, such as ranibizumab (Smith et al., 2009; Konstantinidis et al., 2010). Although these reports have raised the question of whether anti-VEGF therapy contributes to the development of RPE tear, the data to date have been anecdotal in nature, making it difficult to assess whether the incidence of RPE tear actually increased in patients receiving intravitreal anti-VEGF therapy. A retrospective analysis of clinical trials of ranibizumab found an overall incidence of RPE tear of 2.4%, which occurs after intravitreal therapy (Cho et al., 2015; Shin et al., 2015). However, a study on an incidence of RPE tear after intravitreal ranibizumab injection for neovascular AMD made no significant difference with the control treatment. This suggests a potential benefit to continuous ranibizumab therapy in patients with neovascular AMD that developed to RPE tear (Cunningham et al., 2011). Currently, there are several mechanisms to explain the development of RPE tear following anti-VEGF injection. One of the most plausible theories is that the anti-VEGF treatment may cause fibrosis contraction of the vascularized tissue underneath the RPE, ripping the overlying RPE (Spaide, 2009) and thus, change of retina during treatment should be closely monitored. In addition, we should take caution in explaining the significant signal, as one of the complications of advanced neovascular AMD.

Glaucoma is currently the leading cause of irreversible blindness worldwide (Quigley, 2006; Miraftabi et al., 2020) due to elevated intraocular pressure (Blumberg et al., 2015). Clinical ophthalmologists are also concerned about the increase of intraocular pressure after the administration of anti-VEGF drugs. A retrospective study estimated the risk of glaucoma or sustained ocular hypertension related to anti-VEGF treatment for neovascular AMD, and found that the rate of injection and lens status are associated with intraocular pressure (Wingard et al., 2019). As the zonular system attached to the lens is fragile, the presumption is that the anterior chamber volume compresses with anterior movement of the lens and iris and thus, may strain the outflow apparatus, and cause increase in intraocular pressure (Kerimoglu et al., 2015). Therefore, eye monitoring should be closely observed for at least 30 min after administration of anti-VEGF drugs.

Dry eye syndrome is defined as chronic inflammatory condition on the ocular surface. Typical symptoms include burning and itchiness, gritty sensation, tearing, redness of the conjunctiva, foreign body sensation, and blurred vision. These have been associated with several clinical markers including tear hyperosmolarity, elevated inflammatory markers, and abnormal tear production (Calonge et al., 2010). Dry eye syndrome is a common complaint among patients undergoing prolonged treatment with anti-VEGF drugs due to repeated exposure to preservatives contained in antibiotic eye drops that causes eye discomfort (Ayaki et al., 2012). As hyperosmolarity is a key event in the pathology of dry eye, it should be used as a marker for testing, diagnosis, and follow-up for chronic ocular treatments to identify the presence of dry eye syndrome (Versura et al., 2010). To prevent any progression, one should focus on measuring and treating the symptoms of tear hyperosmolarity, as initial treatment.

Based on the four-grid table of ratio imbalance, the information about drugs and its AEs are comprehensively considered and the relationship between them is objectively reflected. This provides strong support for the monitoring of adverse drug reactions and rational clinical use of drugs. But spontaneous reporting system has its own limitations. Omission or misstatement could exist and repeated reporting bias. Besides, the number of AE reports is influenced by the time of drug launch, country and region, and the severity of AE. And although brolucizumab in this study has fewer safety signals than the other two drugs, it cannot be inferred that brolucizumab is safer to use. In addition, some AEs may be caused by intraocular injection. Although some have high signal values, we have not analyzed them because no evidence has been found to date, and further research may be needed. Therefore, causality cannot be confirmed based on the FAERS data alone. Moreover, organization of AE reports, rectification of disproportionality analysis at the SMQ level, and application of stricter signal threshold (IC-2SD ≥ 1.5) were performed to address the limitations of FAERS (Huang et al., 2020). As a conclusion, this study only suggests the possible AEs and intensity of anti-VEGF drugs, and further clinical studies are needed for higher-level evidence.

## Conclusion

In conclusion, our results suggest that ocular AEs associated with anti-VEGF drugs varies, and clinicians should consider specific risk factors based on patients’ condition. Our study design does not allow any causality proof, and even though appropriate clinically performed assessment is necessary to validate our claims, it is a step toward understanding the safety profile of anti-VEGF drugs for optimal use.

## Data Availability

The original contributions presented in the study are included in the article/Supplementary Materials, further inquiries can be directed to the corresponding author.
