# Supplementary material for: Ocular adverse events associated with anti-VEGF therapy: A pharmacovigilance study of the FDA adverse event reporting system (FAERS)
Source: Front Pharmacol. 2022 Nov 18;13:1017889. doi: 10.3389/fphar.2022.1017889 (PMC9716077; doi:10.3389/fphar.2022.1017889)
Supplement: Supplementary file 1 [file Table3.DOCX]

**Supplementary Data**

# Table S1. All moderate and strong signal ocular adverse events based on IC-2SD numerical size ordering of Ranizumab.

| **PT** | **N** | **ROR (95%CI)** | **IC (IC-2SD)** |
| --- | --- | --- | --- |
| Retinal pigment epithelial tear | 356 | 836.54 (706.72, 990.22) | 7.39 (7.19) |
| Choroidal neovascularisation | 288 | 591.21 (498.88, 700.62) | 7.13 (6.92) |
| Detachment of retinal pigment epithelium | 292 | 440.01 (376.16, 514.69) | 7.03 (6.82) |
| Age-related macular degeneration | 239 | 329.36 (279.83, 387.66) | 6.77 (6.55) |
| Retinal oedema | 274 | 212.79 (184.72, 245.11) | 6.60 (6.40) |
| Subretinal fluid | 162 | 362.28 (296.11, 443.24) | 6.53 (6.26) |
| Diabetic retinal oedema | 136 | 543.44 (426.74, 692.06) | 6.49 (6.19) |
| Vitreous haemorrhage | 333 | 133.70 (118.43, 150.94) | 6.32 (6.14) |
| Endophthalmitis | 590 | 109.11 (99.74, 119.36) | 6.26 (6.13) |
| Retinal haemorrhage | 660 | 97.72 (89.83, 106.31) | 6.16 (6.04) |
| Retinal scar | 122 | 377.92 (298.95, 477.77) | 6.30 (5.98) |
| Polypoidal choroidal vasculopathy | 100 | 928.35 (667.87, 1290.42) | 6.27 (5.90) |
| Visual acuity reduced | 2437 | 71.38 (68.27, 74.63) | 5.78 (5.72) |
| Eye haemorrhage | 744 | 63.18 (58.48, 68.25) | 5.67 (5.56) |
| Metamorphopsia | 150 | 90.53 (76.06, 107.75) | 5.67 (5.42) |
| Retinal exudates | 115 | 110.45 (90.24, 135.17) | 5.67 (5.38) |
| Macular oedema | 331 | 58.96 (52.58, 66.11) | 5.50 (5.33) |
| Macular hole | 117 | 95.96 (78.73, 116.97) | 5.59 (5.30) |
| Vitreous floaters | 401 | 50.89 (45.89, 56.44) | 5.36 (5.21) |
| Retinal thickening | 60 | 679.48 (461.54, 1000.33) | 5.66 (5.19) |
| Cystoid macular oedema | 120 | 78.12 (64.44, 94.71) | 5.44 (5.16) |
| Retinopathy of prematurity | 78 | 133.95 (104.35, 171.95) | 5.51 (5.15) |
| Subretinal fibrosis | 59 | 375.82 (268.40, 526.22) | 5.56 (5.11) |
| Retinal tear | 132 | 62.55 (52.18, 74.98) | 5.30 (5.03) |
| Retinal cyst | 53 | 465.52 (320.68, 675.77) | 5.47 (4.99) |
| Ocular hypertension | 102 | 68.17 (55.42, 83.85) | 5.25 (4.95) |
| Vitritis | 97 | 67.92 (54.93, 83.99) | 5.22 (4.91) |
| Retinal vein occlusion | 149 | 50.30 (42.48, 59.55) | 5.13 (4.89) |
| Retinal neovascularisation | 48 | 274.69 (193.32, 390.29) | 5.27 (4.79) |
| Vitreous disorder | 56 | 119.38 (89.21, 159.75) | 5.17 (4.75) |
| Retinal vascular disorder | 59 | 93.95 (71.15, 124.07) | 5.10 (4.70) |
| Neovascular age-related macular degeneration | 56 | 106.07 (79.51, 141.50) | 5.12 (4.70) |
| Retinal disorder | 114 | 44.77 (36.95, 54.24) | 4.93 (4.65) |
| Retinal ischaemia | 56 | 89.16 (67.10, 118.47) | 5.03 (4.62) |
| Retinal degeneration | 65 | 60.24 (46.57, 77.92) | 4.90 (4.53) |
| Retinal detachment | 261 | 30.46 (26.86, 34.53) | 4.69 (4.50) |
| Macular degeneration | 282 | 28.06 (24.87, 31.65) | 4.59 (4.42) |
| Macular scar | 36 | 199.18 (135.46, 292.88) | 4.88 (4.35) |
| Maculopathy | 100 | 34.45 (28.12, 42.20) | 4.62 (4.32) |
| Vitreous opacities | 48 | 64.50 (47.76, 87.12) | 4.73 (4.30) |
| Vitrectomy | 45 | 67.60 (49.52, 92.28) | 4.71 (4.26) |
| Vitreous detachment | 72 | 37.96 (29.87, 48.25) | 4.58 (4.23) |
| Anterior chamber inflammation | 41 | 75.36 (54.27, 104.64) | 4.69 (4.22) |
| Blindness unilateral | 341 | 22.62 (20.28, 25.24) | 4.33 (4.17) |
| Macular fibrosis | 42 | 60.07 (43.62, 82.73) | 4.59 (4.13) |
| Dry age-related macular degeneration | 33 | 126.28 (86.23, 184.92) | 4.68 (4.13) |
| Eye inflammation | 167 | 24.13 (20.65, 28.20) | 4.34 (4.11) |
| Intraocular pressure increased | 310 | 21.58 (19.24, 24.19) | 4.27 (4.10) |
| Cataract nuclear | 36 | 78.31 (55.12, 111.26) | 4.59 (4.09) |
| Retinal artery occlusion | 76 | 29.52 (23.41, 37.21) | 4.37 (4.03) |
| Non-infectious endophthalmitis | 41 | 50.67 (36.74, 69.86) | 4.47 (4.01) |
| Anterior chamber cell | 37 | 61.15 (43.47, 86.02) | 4.50 (4.01) |
| Blindness | 736 | 18.51 (17.18, 19.95) | 4.08 (3.97) |
| Hypopyon | 41 | 44.51 (32.34, 61.26) | 4.39 (3.93) |
| Eye oedema | 66 | 28.48 (22.22, 36.51) | 4.28 (3.92) |
| Lens disorder | 29 | 100.38 (67.39, 149.53) | 4.48 (3.91) |
| Visual field defect | 179 | 19.54 (16.82, 22.70) | 4.09 (3.87) |
| Eye infection | 160 | 19.64 (16.76, 23.01) | 4.08 (3.85) |
| Retinal depigmentation | 27 | 112.60 (74.20, 170.88) | 4.43 (3.84) |
| Eye pain | 698 | 16.21 (15.01, 17.50) | 3.90 (3.79) |
| Corneal abrasion | 41 | 33.83 (24.65, 46.42) | 4.19 (3.73) |
| Macular ischaemia | 23 | 205.27 (126.46, 333.18) | 4.37 (3.70) |
| Suspected transmission of an infectious agent via product | 35 | 38.23 (27.11, 53.91) | 4.18 (3.68) |
| Retinal aneurysm | 23 | 134.48 (84.92, 212.98) | 4.30 (3.65) |
| Foreign body sensation in eyes | 85 | 18.67 (15.03, 23.19) | 3.91 (3.59) |
| Pseudoendophthalmitis | 21 | 190.74 (115.49, 315.03) | 4.25 (3.56) |
| Choroidal haemorrhage | 22 | 105.57 (66.69, 167.14) | 4.20 (3.54) |
| Chorioretinal atrophy | 23 | 70.48 (45.56, 109.03) | 4.13 (3.50) |
| Iridocyclitis | 52 | 19.93 (15.10, 26.30) | 3.83 (3.43) |
| Cataract subcapsular | 25 | 45.26 (30.06, 68.15) | 4.02 (3.43) |
| Conjunctival haemorrhage | 70 | 16.73 (13.18, 21.23) | 3.74 (3.39) |
| Posterior capsule opacification | 19 | 90.31 (55.43, 147.14) | 4.00 (3.31) |
| Retinal vascular thrombosis | 24 | 36.12 (23.87, 54.66) | 3.87 (3.27) |
| Foreign body in eye | 26 | 31.05 (20.89, 46.15) | 3.84 (3.26) |
| Retinal fibrosis | 17 | 392.98 (208.65, 740.13) | 4.06 (3.24) |
| Vitreous haze | 17 | 154.38 (89.70, 265.70) | 3.97 (3.22) |
| Corneal erosion | 22 | 37.93 (24.59, 58.51) | 3.82 (3.20) |
| Retinal vasculitis | 28 | 23.82 (16.30, 34.81) | 3.70 (3.15) |
| Diabetic retinopathy | 51 | 15.25 (11.54, 20.16) | 3.55 (3.14) |
| Hyphaema | 21 | 37.74 (24.22, 58.81) | 3.78 (3.14) |
| Uveitis | 109 | 11.49 (9.50, 13.90) | 3.36 (3.08) |
| Ocular discomfort | 79 | 12.36 (9.89, 15.46) | 3.40 (3.08) |
| Serous retinal detachment | 20 | 37.26 (23.66, 58.69) | 3.73 (3.08) |
| Eye injury | 39 | 16.18 (11.76, 22.26) | 3.52 (3.06) |
| Visual impairment | 1025 | 9.22 (8.65, 9.82) | 3.11 (3.01) |
| Cataract | 430 | 9.41 (8.54, 10.36) | 3.16 (3.01) |
| Glaucoma | 182 | 9.85 (8.50, 11.42) | 3.20 (2.98) |
| Neovascularisation | 15 | 90.80 (52.41, 157.32) | 3.74 (2.96) |
| Eye discharge | 77 | 10.87 (8.67, 13.63) | 3.24 (2.91) |
| Blindness transient | 80 | 10.62 (8.51, 13.26) | 3.22 (2.89) |
| Corneal oedema | 35 | 14.46 (10.33, 20.24) | 3.37 (2.88) |
| Photopsia | 68 | 10.81 (8.50, 13.75) | 3.21 (2.86) |
| Eye disorder | 267 | 8.61 (7.63, 9.73) | 3.03 (2.85) |
| Cerebral infarction | 227 | 8.55 (7.49, 9.75) | 3.01 (2.82) |
| Cataract traumatic | 13 | 244.81 (126.31, 474.51) | 3.70 (2.80) |
| Ocular hyperaemia | 308 | 8.17 (7.29, 9.15) | 2.96 (2.79) |
| Normal tension glaucoma | 13 | 118.03 (64.55, 215.84) | 3.62 (2.78) |
| Glare | 16 | 33.76 (20.35, 56.01) | 3.50 (2.77) |
| Anterior chamber disorder | 14 | 55.61 (32.03, 96.58) | 3.55 (2.77) |
| Retinal injury | 18 | 24.09 (15.01, 38.66) | 3.41 (2.74) |
| Open angle glaucoma | 17 | 26.93 (16.53, 43.87) | 3.43 (2.74) |
| Ophthalmological examination abnormal | 13 | 75.98 (42.41, 136.10) | 3.55 (2.72) |
| Retinopathy proliferative | 13 | 74.27 (41.50, 132.93) | 3.54 (2.72) |
| Lacrimation increased | 183 | 8.04 (6.95, 9.31) | 2.92 (2.71) |
| Eye infection bacterial | 15 | 34.51 (20.45, 58.24) | 3.45 (2.70) |
| Keratic precipitates | 14 | 44.49 (25.76, 76.84) | 3.48 (2.70) |
| Tachyphylaxis | 15 | 33.02 (19.58, 55.67) | 3.43 (2.68) |
| Visual acuity tests abnormal | 13 | 61.20 (34.42, 108.83) | 3.50 (2.68) |
| Staphylococcus test positive | 25 | 14.79 (9.93, 22.01) | 3.25 (2.67) |
| Macular cyst | 12 | 135.58 (71.71, 256.34) | 3.54 (2.66) |
| Retinal drusen | 12 | 127.11 (67.51, 239.31) | 3.54 (2.66) |
| Retinoschisis | 12 | 127.11 (67.51, 239.31) | 3.54 (2.66) |
| Vitreous adhesions | 12 | 122.02 (64.98, 229.16) | 3.53 (2.65) |
| Intercepted medication error | 61 | 9.01 (6.99, 11.60) | 2.97 (2.60) |
| Corneal disorder | 28 | 12.57 (8.64, 18.30) | 3.14 (2.60) |
| Abnormal sensation in eye | 45 | 9.80 (7.29, 13.17) | 3.02 (2.59) |
| Iritis | 33 | 10.97 (7.77, 15.49) | 3.06 (2.56) |
| Choroidal detachment | 15 | 25.01 (14.89, 42.00) | 3.29 (2.56) |
| Eye infection intraocular | 11 | 114.13 (59.34, 219.52) | 3.42 (2.51) |
| Anterior chamber flare | 11 | 73.59 (39.10, 138.50) | 3.35 (2.47) |
| Macular detachment | 11 | 76.61 (40.64, 144.43) | 3.36 (2.47) |
| Cytomegalovirus chorioretinitis | 20 | 13.37 (8.57, 20.84) | 3.05 (2.41) |
| Intercepted product storage error | 23 | 11.78 (7.79, 17.82) | 3.00 (2.40) |
| Cataract operation | 41 | 8.46 (6.21, 11.52) | 2.82 (2.38) |
| Punctate keratitis | 14 | 21.00 (12.30, 35.84) | 3.15 (2.38) |
| Choroidal dystrophy | 10 | 141.22 (70.07, 284.59) | 3.33 (2.37) |
| Optic atrophy | 22 | 11.61 (7.61, 17.72) | 2.97 (2.36) |
| Transient ischaemic attack | 239 | 6.04 (5.31, 6.87) | 2.54 (2.35) |
| Charles Bonnet syndrome | 10 | 105.91 (53.58, 209.36) | 3.30 (2.35) |
| Iris neovascularisation | 10 | 86.17 (44.07, 168.46) | 3.27 (2.34) |
| Corneal lesion | 11 | 37.28 (20.21, 68.78) | 3.19 (2.32) |
| Ocular vascular disorder | 16 | 14.77 (8.98, 24.28) | 3.01 (2.30) |
| Hypotony of eye | 12 | 24.11 (13.51, 43.04) | 3.09 (2.27) |
| Cerebrovascular accident | 1173 | 5.33 (5.02, 5.65) | 2.33 (2.24) |
| Intraocular pressure decreased | 12 | 20.54 (11.53, 36.59) | 3.01 (2.20) |
| Eye infection staphylococcal | 10 | 36.84 (19.39, 70.01) | 3.09 (2.19) |
| Retinal vascular occlusion | 13 | 16.48 (9.49, 28.64) | 2.95 (2.16) |
| Vision blurred | 623 | 4.95 (4.57, 5.37) | 2.25 (2.14) |
| Angle closure glaucoma | 27 | 8.25 (5.64, 12.07) | 2.69 (2.14) |
| Visual acuity reduced transiently | 10 | 31.38 (16.57, 59.44) | 3.04 (2.14) |
| Macular pseudohole | 9 | 508.36 (201.77, 1280.81) | 3.27 (2.14) |
| Deafness | 148 | 5.36 (4.55, 6.30) | 2.36 (2.12) |
| Keratitis | 22 | 8.72 (5.72, 13.30) | 2.69 (2.08) |
| Intraocular melanoma | 9 | 40.49 (20.53, 79.84) | 3.01 (2.06) |
| Deposit eye | 9 | 35.74 (18.18, 70.28) | 2.98 (2.03) |
| Eye irritation | 203 | 4.84 (4.21, 5.56) | 2.23 (2.02) |
| Concomitant disease progression | 22 | 8.11 (5.32, 12.37) | 2.62 (2.01) |
| Giant cell arteritis | 15 | 10.90 (6.53, 18.17) | 2.73 (2.00) |
| Corneal exfoliation | 8 | 131.18 (60.29, 285.42) | 3.06 (2.00) |
| Death | 3773 | 4.80 (4.63, 4.97) | 2.03 (1.98) |
| Central vision loss | 8 | 79.74 (37.84, 168.04) | 3.01 (1.98) |
| Blepharitis | 22 | 7.77 (5.10, 11.84) | 2.57 (1.96) |
| Intraocular pressure test abnormal | 11 | 15.84 (8.69, 28.87) | 2.81 (1.96) |
| Posterior capsule rupture | 8 | 63.54 (30.46, 132.53) | 2.98 (1.96) |
| Retinal pallor | 8 | 369.70 (148.69, 919.22) | 3.11 (1.96) |
| Cutis laxa | 9 | 26.60 (13.61, 52.00) | 2.88 (1.94) |
| Lens dislocation | 8 | 53.51 (25.82, 110.89) | 2.95 (1.94) |
| Subretinal haematoma | 8 | 451.86 (174.32, 1171.28) | 3.12 (1.94) |
| Vascular dementia | 11 | 14.49 (7.96, 26.38) | 2.75 (1.91) |
| Lenticular opacities | 9 | 23.34 (11.96, 45.54) | 2.83 (1.90) |
| Photophobia | 78 | 4.93 (3.94, 6.16) | 2.22 (1.89) |
| Eye pruritus | 106 | 4.68 (3.87, 5.67) | 2.16 (1.88) |
| Disease recurrence | 101 | 4.59 (3.78, 5.59) | 2.13 (1.85) |
| Asthenopia | 28 | 6.24 (4.30, 9.07) | 2.39 (1.85) |
| Intraocular lens implant | 9 | 20.24 (10.40, 39.42) | 2.77 (1.84) |
| Left ventricular failure | 22 | 6.85 (4.50, 10.44) | 2.44 (1.83) |
| Colour blindness acquired | 9 | 18.91 (9.72, 36.78) | 2.74 (1.81) |
| Retinal pigmentation | 9 | 18.45 (9.49, 35.88) | 2.73 (1.80) |
| Conjunctival hyperaemia | 22 | 6.64 (4.36, 10.11) | 2.40 (1.79) |
| Ocular ischaemic syndrome | 7 | 101.66 (45.15, 228.90) | 2.88 (1.77) |
| Detachment of macular retinal pigment epithelium | 7 | 197.68 (82.56, 473.33) | 2.93 (1.76) |
| Macular pigmentation | 7 | 80.87 (36.42, 179.56) | 2.86 (1.76) |
| Pupillary disorder | 10 | 13.20 (7.05, 24.74) | 2.63 (1.75) |
| Cataract cortical | 7 | 46.21 (21.31, 100.19) | 2.78 (1.71) |
| Panophthalmitis | 7 | 42.36 (19.59, 91.59) | 2.76 (1.70) |
| Conjunctivitis | 63 | 4.38 (3.42, 5.62) | 2.05 (1.68) |
| Eyelid pain | 11 | 10.19 (5.61, 18.51) | 2.51 (1.67) |
| Concomitant disease aggravated | 33 | 4.99 (3.54, 7.03) | 2.15 (1.65) |
| Bronchial carcinoma | 10 | 11.08 (5.92, 20.73) | 2.52 (1.64) |
| Injury corneal | 8 | 17.84 (8.81, 36.10) | 2.62 (1.64) |
| Iris adhesions | 8 | 17.76 (8.77, 35.94) | 2.62 (1.64) |
| Eye swelling | 124 | 3.83 (3.21, 4.57) | 1.89 (1.63) |
| Haemorrhagic stroke | 38 | 4.64 (3.37, 6.39) | 2.07 (1.61) |
| Cerebellar infarction | 14 | 7.29 (4.30, 12.36) | 2.35 (1.60) |
| Ulcerative keratitis | 17 | 6.13 (3.80, 9.89) | 2.24 (1.56) |
| Choroidal effusion | 9 | 11.24 (5.81, 21.76) | 2.46 (1.54) |
| Myocardial ischaemia | 67 | 3.88 (3.05, 4.94) | 1.89 (1.53) |
| Aortic aneurysm rupture | 10 | 9.47 (5.06, 17.70) | 2.41 (1.53) |
| Optic ischaemic neuropathy | 19 | 5.59 (3.56, 8.79) | 2.17 (1.52) |
| Optic nerve injury | 10 | 9.41 (5.04, 17.60) | 2.40 (1.52) |
| Hemianopia homonymous | 9 | 10.97 (5.67, 21.24) | 2.44 (1.52) |
| Eye paraesthesia | 6 | 84.71 (35.69, 201.07) | 2.69 (1.51) |
| Exudative retinopathy | 6 | 190.61 (74.58, 487.16) | 2.74 (1.50) |

# Table S2. All moderate and strong signal ocular adverse events based on IC-2SD numerical size ordering of Aflibercept.

| **PT** | **N** | **ROR (95%CI)** | **IC (IC-2SD)** |
| --- | --- | --- | --- |
| Non-infectious endophthalmitis | 274 | 860.68 (712.53, 1039.64) | 7.24 (7.02) |
| Endophthalmitis | 805 | 178.27 (164.51, 193.19) | 6.82 (6.70) |
| Vitrectomy | 190 | 548.23 (448.60, 669.99) | 6.82 (6.56) |
| Vitritis | 237 | 225.79 (194.06, 262.71) | 6.58 (6.37) |
| Blindness transient | 625 | 108.65 (99.62, 118.49) | 6.28 (6.15) |
| Eye inflammation | 585 | 106.96 (97.81, 116.97) | 6.25 (6.12) |
| Anterior chamber cell | 93 | 205.53 (161.96, 260.81) | 5.86 (5.52) |
| Neovascular age-related macular degeneration | 82 | 187.82 (146.17, 241.34) | 5.71 (5.35) |
| Vitreous opacities | 88 | 144.53 (114.27, 182.80) | 5.65 (5.31) |
| Vitreous floaters | 386 | 53.28 (47.96, 59.20) | 5.41 (5.26) |
| Blindness unilateral | 619 | 47.22 (43.45, 51.32) | 5.31 (5.19) |
| Macular hole | 101 | 88.06 (71.35, 108.68) | 5.45 (5.14) |
| Vitreous haemorrhage | 157 | 60.00 (50.86, 70.78) | 5.33 (5.09) |
| Retinal oedema | 107 | 71.84 (58.71, 87.91) | 5.33 (5.03) |
| Retinal haemorrhage | 307 | 44.37 (39.47, 49.88) | 5.17 (5.00) |
| Eye haemorrhage | 455 | 39.77 (36.13, 43.79) | 5.08 (4.94) |
| Subretinal fluid | 66 | 112.94 (86.64, 147.20) | 5.30 (4.91) |
| Anterior chamber inflammation | 60 | 129.29 (97.59, 171.29) | 5.28 (4.87) |
| Hypopyon | 66 | 82.67 (63.81, 107.10) | 5.12 (4.75) |
| Intraocular pressure increased | 402 | 31.09 (28.10, 34.41) | 4.76 (4.61) |
| Age-related macular degeneration | 65 | 66.08 (51.08, 85.49) | 4.97 (4.60) |
| Detachment of retinal pigment epithelium | 63 | 61.35 (47.27, 79.61) | 4.90 (4.52) |
| Visual acuity reduced transiently | 41 | 173.83 (122.37, 246.93) | 5.00 (4.50) |
| Diabetic retinal oedema | 44 | 111.10 (80.35, 153.61) | 4.93 (4.46) |
| Inadequate aseptic technique in use of product | 38 | 180.36 (125.05, 260.13) | 4.93 (4.41) |
| Dry age-related macular degeneration | 38 | 164.86 (114.75, 236.86) | 4.91 (4.39) |
| Retinal pigment epithelial tear | 55 | 58.67 (44.42, 77.50) | 4.78 (4.37) |
| Polypoidal choroidal vasculopathy | 36 | 167.98 (115.67, 243.94) | 4.85 (4.32) |
| Uveitis | 222 | 26.30 (22.97, 30.11) | 4.49 (4.29) |
| Keratic precipitates | 36 | 144.85 (100.34, 209.09) | 4.81 (4.29) |
| Ocular hypertension | 62 | 42.87 (33.10, 55.53) | 4.62 (4.24) |
| Retinal vasculitis | 51 | 49.29 (37.00, 65.65) | 4.61 (4.19) |
| Intraocular lens implant | 36 | 100.45 (70.41, 143.30) | 4.70 (4.19) |
| Hypotony of eye | 36 | 87.29 (61.41, 124.08) | 4.64 (4.14) |
| Blindness | 747 | 20.58 (19.10, 22.17) | 4.23 (4.12) |
| Metamorphopsia | 62 | 36.83 (28.47, 47.64) | 4.49 (4.12) |
| Vitreous haze | 29 | 365.83 (228.87, 584.76) | 4.73 (4.10) |
| Retinal artery occlusion | 74 | 31.31 (24.76, 39.58) | 4.42 (4.08) |
| Eye infection bacterial | 33 | 90.25 (62.45, 130.41) | 4.57 (4.05) |
| Vitreal cells | 28 | 361.41 (224.49, 581.84) | 4.68 (4.05) |
| Iritis | 76 | 28.42 (22.56, 35.80) | 4.33 (4.00) |
| Vitreous disorder | 34 | 72.32 (50.58, 103.42) | 4.51 (4.00) |
| Intraocular pressure decreased | 34 | 68.64 (48.05, 98.05) | 4.49 (3.97) |
| Device use issue | 136 | 21.64 (18.22, 25.70) | 4.18 (3.93) |
| Macular oedema | 119 | 21.33 (17.75, 25.63) | 4.14 (3.87) |
| Visual acuity reduced | 636 | 16.99 (15.68, 18.41) | 3.97 (3.85) |
| Choroidal neovascularisation | 38 | 42.12 (30.28, 58.59) | 4.31 (3.83) |
| Ocular procedural complication | 25 | 106.72 (69.54, 163.77) | 4.34 (3.73) |
| Iridocyclitis | 59 | 24.81 (19.11, 32.21) | 4.10 (3.72) |
| Anterior chamber flare | 23 | 199.41 (123.80, 321.20) | 4.37 (3.71) |
| Proteus infection | 28 | 60.00 (40.62, 88.64) | 4.26 (3.69) |
| Eye pain | 590 | 14.83 (13.65, 16.11) | 3.78 (3.66) |
| Corneal oedema | 53 | 24.26 (18.42, 31.95) | 4.04 (3.64) |
| Cataract operation | 84 | 19.29 (15.51, 23.98) | 3.94 (3.63) |
| Macular fibrosis | 30 | 45.25 (31.18, 65.67) | 4.17 (3.63) |
| Eye infection | 127 | 16.85 (14.12, 20.12) | 3.86 (3.60) |
| Ocular discomfort | 99 | 17.03 (13.93, 20.81) | 3.83 (3.54) |
| Eye infection staphylococcal | 22 | 96.88 (61.58, 152.41) | 4.18 (3.53) |
| Retinal exudates | 34 | 30.79 (21.79, 43.51) | 4.01 (3.51) |
| Retinal scar | 25 | 52.95 (35.12, 79.83) | 4.10 (3.51) |
| Amaurosis fugax | 30 | 36.12 (24.96, 52.27) | 4.04 (3.50) |
| Eye laser surgery | 24 | 57.65 (37.86, 87.80) | 4.10 (3.49) |
| Foreign body sensation in eyes | 74 | 17.64 (13.99, 22.24) | 3.81 (3.47) |
| Multiple use of single-use product | 68 | 18.23 (14.31, 23.22) | 3.83 (3.47) |
| Retinal thickening | 20 | 130.54 (80.19, 212.51) | 4.14 (3.45) |
| Retinal detachment | 119 | 14.58 (12.15, 17.50) | 3.67 (3.40) |
| Colitis microscopic | 60 | 17.24 (13.33, 22.29) | 3.73 (3.35) |
| Eye operation | 49 | 17.99 (13.53, 23.92) | 3.71 (3.30) |
| Refusal of treatment by patient | 89 | 13.74 (11.13, 16.97) | 3.56 (3.25) |
| Retinal vein occlusion | 48 | 16.49 (12.37, 21.98) | 3.61 (3.20) |
| Corneal abrasion | 28 | 24.67 (16.89, 36.03) | 3.73 (3.18) |
| Eye infection intraocular | 16 | 201.70 (113.80, 357.51) | 3.94 (3.15) |
| Intra-ocular injection complication | 16 | 170.67 (97.44, 298.94) | 3.92 (3.14) |
| Anterior chamber disorder | 17 | 75.44 (45.44, 125.24) | 3.84 (3.12) |
| Retinal degeneration | 26 | 24.50 (16.54, 36.29) | 3.68 (3.11) |
| Prothrombin level decreased | 23 | 27.80 (18.29, 42.28) | 3.68 (3.08) |
| Suspected product quality issue | 22 | 30.29 (19.72, 46.53) | 3.70 (3.08) |
| Death | 7305 | 13.54 (13.15, 13.94) | 3.11 (3.07) |
| Conjunctival haemorrhage | 53 | 13.70 (10.43, 17.99) | 3.44 (3.04) |
| Retinal vascular occlusion | 21 | 29.65 (19.11, 46.00) | 3.65 (3.02) |
| Intra-ocular injection | 15 | 756.34 (347.35, 1646.92) | 3.93 (3.02) |
| Vitreous detachment | 31 | 17.07 (11.94, 24.41) | 3.48 (2.96) |
| Foreign body in eye | 20 | 25.68 (16.40, 40.22) | 3.53 (2.89) |
| Toxic anterior segment syndrome | 20 | 24.17 (15.45, 37.83) | 3.49 (2.85) |
| Visual impairment | 837 | 8.13 (7.59, 8.72) | 2.94 (2.84) |
| Eye oedema | 32 | 14.62 (10.29, 20.77) | 3.34 (2.84) |
| Retinal ischaemia | 18 | 27.89 (17.37, 44.79) | 3.50 (2.82) |
| Retinal disorder | 34 | 13.67 (9.72, 19.21) | 3.30 (2.81) |
| Macular degeneration | 91 | 9.44 (7.67, 11.62) | 3.09 (2.78) |
| Pseudoendophthalmitis | 13 | 112.65 (62.04, 204.53) | 3.62 (2.78) |
| Necrotising fasciitis | 38 | 12.32 (8.93, 17.00) | 3.23 (2.76) |
| Retinal vascular disorder | 17 | 26.05 (16.01, 42.38) | 3.42 (2.72) |
| Macular scar | 13 | 62.69 (35.32, 111.27) | 3.51 (2.69) |
| Sudden visual loss | 13 | 55.46 (31.35, 98.10) | 3.47 (2.67) |
| Rhegmatogenous retinal detachment | 12 | 121.00 (64.79, 225.97) | 3.53 (2.66) |
| Serous retinal detachment | 14 | 27.83 (16.27, 47.61) | 3.29 (2.53) |
| Induration | 22 | 13.85 (9.07, 21.16) | 3.13 (2.52) |
| Klebsiella infection | 32 | 9.83 (6.93, 13.95) | 2.94 (2.43) |
| Cataract | 261 | 6.14 (5.43, 6.94) | 2.56 (2.38) |
| Lacrimation increased | 136 | 6.48 (5.47, 7.68) | 2.62 (2.37) |
| Corneal disorder | 23 | 11.21 (7.42, 16.95) | 2.96 (2.36) |
| Product impurity | 10 | 81.54 (41.98, 158.40) | 3.27 (2.34) |
| Multiple-drug resistance | 21 | 11.56 (7.50, 17.81) | 2.95 (2.32) |
| Retinal deposits | 10 | 59.62 (31.05, 114.49) | 3.21 (2.30) |
| Photophobia | 90 | 6.22 (5.05, 7.66) | 2.54 (2.23) |
| Injury associated with device | 93 | 6.05 (4.93, 7.42) | 2.50 (2.20) |
| Eye disorder | 160 | 5.57 (4.76, 6.51) | 2.42 (2.19) |
| Visual field defect | 57 | 6.58 (5.06, 8.54) | 2.57 (2.19) |
| Glaucoma | 100 | 5.83 (4.79, 7.11) | 2.46 (2.17) |
| Retinal tear | 21 | 9.79 (6.36, 15.08) | 2.79 (2.17) |
| Ocular hyperaemia | 185 | 5.29 (4.58, 6.12) | 2.35 (2.14) |
| Diabetic retinopathy | 26 | 8.35 (5.67, 12.30) | 2.70 (2.14) |
| Panophthalmitis | 9 | 60.86 (30.57, 121.14) | 3.10 (2.14) |
| Cardiac myxoma | 9 | 51.98 (26.24, 102.96) | 3.07 (2.12) |
| Laser therapy | 9 | 50.41 (25.47, 99.75) | 3.06 (2.11) |
| Retinal cyst | 9 | 48.92 (24.74, 96.73) | 3.06 (2.10) |
| Chorioretinitis | 10 | 25.79 (13.68, 48.63) | 2.97 (2.08) |
| Corneal opacity | 14 | 12.58 (7.41, 21.38) | 2.81 (2.06) |
| Retinal neovascularisation | 9 | 38.99 (19.83, 76.65) | 3.00 (2.06) |
| Maculopathy | 23 | 8.19 (5.42, 12.36) | 2.64 (2.05) |
| Vision blurred | 537 | 4.64 (4.26, 5.06) | 2.16 (2.04) |
| Eye injury | 20 | 8.90 (5.72, 13.85) | 2.68 (2.04) |
| Pterygium | 9 | 36.16 (18.42, 70.98) | 2.98 (2.04) |
| Superior vena cava syndrome | 10 | 22.82 (12.12, 42.96) | 2.92 (2.03) |
| Retinal depigmentation | 9 | 35.64 (18.16, 69.95) | 2.98 (2.03) |
| Amaurosis | 11 | 16.90 (9.27, 30.79) | 2.84 (2.00) |
| Retinitis | 11 | 16.85 (9.25, 30.70) | 2.84 (2.00) |
| Lens disorder | 9 | 29.88 (15.28, 58.45) | 2.92 (1.98) |
| Injection site discomfort | 45 | 5.82 (4.33, 7.81) | 2.38 (1.95) |
| Corneal erosion | 10 | 18.06 (9.62, 33.91) | 2.81 (1.92) |
| Cystoid macular oedema | 15 | 9.35 (5.61, 15.57) | 2.60 (1.88) |
| Proteinuria | 74 | 4.84 (3.85, 6.09) | 2.19 (1.85) |
| Abnormal sensation in eye | 27 | 6.36 (4.35, 9.30) | 2.40 (1.85) |
| Fournier's gangrene | 14 | 9.73 (5.73, 16.51) | 2.61 (1.85) |
| Eye discharge | 37 | 5.62 (4.07, 7.78) | 2.31 (1.84) |
| Retinal pigment epitheliopathy | 9 | 20.12 (10.35, 39.14) | 2.77 (1.84) |
| Alpha haemolytic streptococcal infection | 10 | 14.55 (7.77, 27.27) | 2.69 (1.81) |
| Bacterial endophthalmitis | 7 | 215.61 (90.04, 516.27) | 2.93 (1.77) |
| Conjunctival scar | 7 | 80.85 (36.58, 178.71) | 2.86 (1.77) |
| Cataract subcapsular | 9 | 16.80 (8.66, 32.62) | 2.69 (1.76) |
| Product contamination | 17 | 7.39 (4.58, 11.93) | 2.43 (1.75) |
| Choroidal detachment | 9 | 16.05 (8.27, 31.13) | 2.66 (1.74) |
| Open globe injury | 8 | 22.86 (11.27, 46.38) | 2.72 (1.74) |
| Cardiac ventricular thrombosis | 9 | 15.40 (7.94, 29.87) | 2.64 (1.72) |
| Iris vascular disorder | 7 | 485.12 (175.90, 1337.94) | 2.96 (1.71) |
| Retinal operation | 7 | 40.01 (18.58, 86.17) | 2.75 (1.69) |
| Necrotising retinitis | 8 | 19.45 (9.61, 39.38) | 2.66 (1.68) |
| Syringe issue | 48 | 4.55 (3.42, 6.04) | 2.07 (1.66) |
| Tachyphylaxis | 8 | 18.64 (9.21, 37.70) | 2.64 (1.66) |
| Puncture site haemorrhage | 9 | 13.60 (7.02, 26.35) | 2.57 (1.65) |
| Posterior capsule opacification | 7 | 32.61 (15.21, 69.90) | 2.70 (1.65) |
| Conjunctival hyperaemia | 19 | 6.24 (3.97, 9.81) | 2.29 (1.64) |
| Deposit eye | 7 | 29.85 (13.95, 63.87) | 2.68 (1.63) |
| Fear of injection | 24 | 5.44 (3.64, 8.14) | 2.20 (1.62) |
| Blepharitis | 17 | 6.53 (4.04, 10.53) | 2.31 (1.62) |
| Retinal injury | 9 | 12.83 (6.62, 24.84) | 2.54 (1.62) |
| Blood lactic acid increased | 23 | 5.49 (3.64, 8.28) | 2.20 (1.61) |
| Clostridial infection | 28 | 5.05 (3.48, 7.33) | 2.14 (1.60) |
| Swelling of eyelid | 15 | 6.76 (4.06, 11.25) | 2.30 (1.58) |
| Neutrophilia | 24 | 5.21 (3.49, 7.79) | 2.15 (1.57) |
| Intentional dose omission | 36 | 4.54 (3.27, 6.31) | 2.04 (1.56) |
| Photopsia | 28 | 4.79 (3.30, 6.94) | 2.07 (1.53) |
| Jugular vein thrombosis | 11 | 8.33 (4.59, 15.12) | 2.36 (1.52) |
| Superficial injury of eye | 9 | 10.97 (5.67, 21.22) | 2.44 (1.52) |
| Retinal perivascular sheathing | 6 | 87.53 (37.00, 207.10) | 2.69 (1.52) |
| Central vision loss | 6 | 62.76 (26.98, 146.01) | 2.66 (1.51) |
| Retinal drusen | 6 | 61.60 (26.50, 143.19) | 2.66 (1.51) |
| Adenocarcinoma of colon | 10 | 9.15 (4.90, 17.09) | 2.38 (1.50) |
| Paraesthesia mucosal | 6 | 57.35 (24.74, 132.93) | 2.65 (1.50) |

# Table S3. All moderate and strong signal ocular adverse events based on IC-2SD numerical size ordering of Brolucizumab.

| **PT** | **N** | **ROR (95%CI)** | **IC (IC-2SD)** |
| --- | --- | --- | --- |
| Vitreous floaters | 453 | 599.32 (538.71, 666.74) | 7.80 (7.65) |
| Retinal vasculitis | 237 | 2930.41 (2480.17, 3462.39) | 7.70 (7.47) |
| Vitritis | 196 | 1469.04 (1244.90, 1733.54) | 7.37 (7.13) |
| Eye inflammation | 280 | 400.22 (351.81, 455.28) | 7.24 (7.06) |
| Anterior chamber cell | 138 | 3068.82 (2464.67, 3821.06) | 7.01 (6.71) |
| Vitreous opacities | 138 | 2198.07 (1787.29, 2703.25) | 6.98 (6.70) |
| Uveitis | 243 | 248.73 (217.20, 284.83) | 6.83 (6.63) |
| Keratic precipitates | 121 | 10432.45 (7534.69, 14444.65) | 6.87 (6.53) |
| Iritis | 120 | 379.53 (313.67, 459.22) | 6.47 (6.20) |
| Retinal artery occlusion | 110 | 391.11 (320.55, 477.19) | 6.39 (6.10) |
| Retinal vascular occlusion | 88 | 1216.18 (957.74, 1544.37) | 6.34 (6.00) |
| Anterior chamber inflammation | 83 | 1587.91 (1232.35, 2046.06) | 6.29 (5.93) |
| Subretinal fluid | 62 | 839.77 (638.30, 1104.83) | 5.85 (5.46) |
| Metamorphopsia | 65 | 310.83 (240.95, 400.96) | 5.75 (5.37) |
| Visual acuity reduced | 277 | 62.20 (54.85, 70.54) | 5.52 (5.33) |
| Intra-ocular injection complication | 53 | 15686.35 (8830.07, 27866.35) | 5.73 (5.20) |
| Iridocyclitis | 61 | 205.82 (158.73, 266.89) | 5.56 (5.18) |
| Eye pain | 245 | 51.20 (44.84, 58.47) | 5.27 (5.08) |
| Blindness transient | 75 | 87.37 (69.28, 110.19) | 5.32 (4.98) |
| Ocular discomfort | 69 | 94.34 (74.08, 120.14) | 5.31 (4.95) |
| Retinal haemorrhage | 72 | 77.97 (61.55, 98.76) | 5.21 (4.87) |
| Visual impairment | 444 | 39.26 (35.41, 43.53) | 4.91 (4.76) |
| Ocular hyperaemia | 164 | 38.98 (33.25, 45.71) | 4.90 (4.66) |
| Vitreal cells | 37 | 4799.11 (3007.42, 7658.20) | 5.22 (4.64) |
| Retinal perivascular sheathing | 37 | 23310.01 (10381.66, 52338.09) | 5.23 (4.59) |
| Vision blurred | 429 | 34.20 (30.81, 37.97) | 4.73 (4.58) |
| Intraocular pressure increased | 80 | 47.01 (37.58, 58.81) | 4.87 (4.54) |
| Retinal oedema | 38 | 186.47 (134.45, 258.61) | 5.00 (4.53) |
| Eye haemorrhage | 74 | 48.20 (38.20, 60.82) | 4.85 (4.51) |
| Anterior chamber flare | 32 | 2560.48 (1653.01, 3966.14) | 5.02 (4.42) |
| Neovascular age-related macular degeneration | 31 | 463.83 (319.66, 673.03) | 4.90 (4.36) |
| Blindness | 139 | 29.71 (25.02, 35.28) | 4.55 (4.30) |
| Visual acuity reduced transiently | 29 | 891.36 (596.81, 1331.29) | 4.85 (4.28) |
| Non-infectious endophthalmitis | 28 | 289.45 (197.06, 425.16) | 4.71 (4.16) |
| Retinal vein occlusion | 32 | 86.23 (60.63, 122.62) | 4.58 (4.07) |
| Visual field defect | 46 | 42.16 (31.45, 56.53) | 4.47 (4.04) |
| Vitreous haze | 25 | 2285.46 (1407.06, 3712.23) | 4.68 (4.02) |
| Endophthalmitis | 38 | 49.60 (35.94, 68.46) | 4.45 (3.98) |
| Lacrimation increased | 70 | 26.57 (20.94, 33.73) | 4.25 (3.90) |
| Photopsia | 35 | 47.60 (34.03, 66.57) | 4.36 (3.87) |
| Photophobia | 54 | 29.63 (22.61, 38.84) | 4.26 (3.86) |
| Retinal ischaemia | 23 | 285.66 (187.01, 436.37) | 4.46 (3.85) |
| Retinal exudates | 21 | 146.95 (94.96, 227.41) | 4.26 (3.63) |
| Chorioretinitis | 19 | 403.72 (251.92, 646.99) | 4.25 (3.57) |
| Retinal disorder | 22 | 69.21 (45.33, 105.65) | 4.12 (3.51) |
| Foreign body sensation in eyes | 25 | 46.03 (30.97, 68.39) | 4.06 (3.49) |
| Eye disorder | 64 | 17.66 (13.77, 22.65) | 3.78 (3.42) |
| Hypopyon | 18 | 160.07 (99.84, 256.63) | 4.09 (3.41) |
| Blindness unilateral | 40 | 21.84 (15.96, 29.87) | 3.84 (3.38) |
| Reading disorder | 20 | 69.61 (44.67, 108.47) | 4.02 (3.38) |
| Age-related macular degeneration | 18 | 132.58 (82.81, 212.26) | 4.06 (3.38) |
| Detachment of retinal pigment epithelium | 18 | 127.85 (79.88, 204.63) | 4.05 (3.38) |
| Eye irritation | 77 | 15.94 (12.70, 20.01) | 3.70 (3.37) |
| Eye discharge | 27 | 32.37 (22.12, 47.37) | 3.92 (3.37) |
| Conjunctival hyperaemia | 21 | 54.52 (35.39, 84.00) | 3.98 (3.36) |
| Eye pruritus | 49 | 18.70 (14.08, 24.82) | 3.76 (3.35) |
| Vasculitis | 32 | 25.09 (17.68, 35.59) | 3.84 (3.34) |
| Macular oedema | 24 | 32.84 (21.93, 49.16) | 3.84 (3.26) |
| Choroidal neovascularisation | 16 | 133.74 (81.20, 220.29) | 3.92 (3.21) |
| Macular degeneration | 28 | 22.65 (15.59, 32.90) | 3.68 (3.14) |
| Dry age-related macular degeneration | 14 | 402.33 (232.39, 696.55) | 3.85 (3.07) |
| Mydriasis | 29 | 18.23 (12.63, 26.30) | 3.52 (2.99) |
| Asthenopia | 18 | 34.37 (21.58, 54.74) | 3.63 (2.97) |
| Cataract | 62 | 11.47 (8.91, 14.76) | 3.27 (2.90) |
| Dry eye | 46 | 12.48 (9.32, 16.71) | 3.30 (2.88) |
| Superficial injury of eye | 13 | 125.86 (72.41, 218.76) | 3.66 (2.88) |
| Inflammation | 49 | 11.99 (9.04, 15.92) | 3.27 (2.86) |
| Concomitant disease aggravated | 19 | 24.61 (15.65, 38.70) | 3.49 (2.84) |
| Presbyopia | 12 | 215.53 (120.56, 385.32) | 3.62 (2.80) |
| Eye injury | 14 | 48.84 (28.80, 82.83) | 3.54 (2.79) |
| Eye swelling | 44 | 11.69 (8.67, 15.76) | 3.22 (2.78) |
| Vitreous haemorrhage | 14 | 38.10 (22.48, 64.57) | 3.45 (2.70) |
| Anterior chamber fibrin | 11 | 841.95 (440.99, 1607.46) | 3.56 (2.66) |
| Hypoaesthesia eye | 11 | 156.83 (85.83, 286.56) | 3.48 (2.63) |
| Eye infection | 18 | 18.26 (11.47, 29.06) | 3.25 (2.58) |
| Glaucoma | 27 | 12.32 (8.43, 18.01) | 3.12 (2.57) |
| Retinal degeneration | 11 | 79.45 (43.71, 144.43) | 3.39 (2.55) |
| Retinal drusen | 10 | 872.20 (441.82, 1721.84) | 3.44 (2.49) |
| Dyschromatopsia | 10 | 150.38 (79.96, 282.82) | 3.36 (2.48) |
| Vitreous disorder | 10 | 153.02 (81.34, 287.83) | 3.36 (2.48) |
| Retinal vascular disorder | 10 | 118.18 (62.98, 221.77) | 3.34 (2.45) |
| Retinal pigment epithelial tear | 10 | 77.05 (41.18, 144.16) | 3.28 (2.40) |
| Injection site discomfort | 16 | 16.17 (9.88, 26.46) | 3.09 (2.38) |
| Eye oedema | 11 | 38.82 (21.42, 70.38) | 3.22 (2.38) |
| Hypoacusis | 47 | 7.99 (5.98, 10.67) | 2.78 (2.36) |
| Visual acuity tests | 10 | 21805.22 (4775.12, 99571.81) | 3.46 (2.27) |
| Foreign body in eye | 9 | 88.56 (45.72, 171.54) | 3.18 (2.25) |
| Corneal opacity | 9 | 63.07 (32.62, 121.94) | 3.13 (2.20) |
| Serous retinal detachment | 8 | 122.31 (60.51, 247.23) | 3.08 (2.10) |
| Vitreous detachment | 9 | 38.09 (19.74, 73.50) | 3.01 (2.09) |
| Idiopathic orbital inflammation | 8 | 1089.36 (501.48, 2366.40) | 3.16 (2.09) |
| Retinal tear | 9 | 32.64 (16.92, 62.95) | 2.97 (2.05) |
| Corneal oedema | 9 | 31.18 (16.17, 60.14) | 2.95 (2.03) |
| Illness | 23 | 7.84 (5.20, 11.83) | 2.60 (2.01) |
| Cystoid macular oedema | 8 | 38.86 (19.35, 78.03) | 2.90 (1.93) |
| Polypoidal choroidal vasculopathy | 7 | 206.01 (96.42, 440.14) | 2.95 (1.90) |
| Central vision loss | 7 | 586.34 (266.08, 1292.06) | 2.98 (1.89) |
| Intraocular pressure decreased | 7 | 100.96 (47.67, 213.81) | 2.90 (1.87) |
| Vascular occlusion | 8 | 30.20 (15.05, 60.61) | 2.83 (1.86) |
| Retinal infiltrates | 7 | 1270.40 (546.87, 2951.20) | 2.99 (1.85) |
| Retinitis | 7 | 83.30 (39.39, 176.16) | 2.88 (1.85) |
| Ocular hypertension | 7 | 35.49 (16.85, 74.75) | 2.74 (1.71) |
| Posterior capsule opacification | 6 | 217.69 (95.79, 494.71) | 2.77 (1.64) |
| Retinal aneurysm | 6 | 251.18 (110.20, 572.53) | 2.77 (1.64) |
| Subretinal fibrosis | 6 | 196.41 (86.59, 445.50) | 2.76 (1.64) |
| Corneal disorder | 7 | 26.42 (12.55, 55.60) | 2.66 (1.63) |
| Ocular vasculitis | 6 | 637.15 (270.26, 1502.14) | 2.79 (1.62) |
| Keratitis | 7 | 23.49 (11.16, 49.42) | 2.62 (1.60) |
| Macular fibrosis | 6 | 66.64 (29.73, 149.39) | 2.68 (1.58) |
| Papilloedema | 8 | 13.75 (6.86, 27.56) | 2.50 (1.54) |
| Maculopathy | 7 | 19.36 (9.20, 40.71) | 2.55 (1.53) |
| Scleritis | 6 | 41.40 (18.51, 92.59) | 2.61 (1.51) |
